# Eloquent Lower Grade Gliomas, a Highly Vulnerable Cohort: Assessment of Patients’ Functional Outcome After Surgery Based on the LoG-Glio Registry

**DOI:** 10.3389/fonc.2022.845992

**Published:** 2022-03-03

**Authors:** Jan Coburger, Julia Onken, Stefan Rueckriegel, Christian von der Brelie, Minou Nadji-Ohl, Marie-Therese Forster, Rüdiger Gerlach, Meike Unteroberdörster, Constantin Roder, Katja Kniese, Stefan Schommer, Dietrich Rothenbacher, Gabriele Nagel, Christian Rainer Wirtz, Ralf-Ingo Ernestus, Arya Nabavi, Marcos Tatagiba, Marcus Czabanka, Oliver Ganslandt, Veit Rohde, Mario Löhr, Peter Vajkoczy, Andrej Pala

**Affiliations:** ^1^Department of Neurosurgery, University of Ulm, Günzburg, Germany; ^2^Department of Neurosurgery, Charité - University of Berlin, Berlin, Germany; ^3^Department of Neurosurgery, University of Würzburg, Würzburg, Germany; ^4^Department of Neurosurgery, University of Göttingen, Göttingen, Germany; ^5^Department of Neurosurgery, Katharinenhospital Stuttgart, Stuttgart, Germany; ^6^Department of Neurosurgery, University of Frankfurt, Frankfurt am Main, Germany; ^7^Department of Neurosurgery, Helios Hospital Erfurt, Erfurt, Germany; ^8^Department of Neurosurgery, University of Tübingen, Tübingen, Germany; ^9^Department of Neurosurgery, KRH Klinikum Region Hannover, Hannover, Germany; ^10^Institute of Epidemiology and Medical Biometry, University of Ulm, Ulm, Germany

**Keywords:** LGG, neurological deficit, awake surgery, iMRI = intraoperative MRI, iUS = intraoperative ultrasound, intraoperative monitoring (IOM), eloquent area tumours, eloquent area surgery

## Abstract

Majority of lower grade glioma (LGG) are located eloquently rendering surgical resection challenging. Aim of our study was to assess rate of permanent deficits and its predisposing risk factors. We retrieved 83 patients harboring an eloquently located LGGs from the prospective LoG-Glio Database. Patients without surgery or incomplete postoperative data were excluded. Sign rank test, explorative correlations by Spearman ρ and multivariable regression for new postoperative deficits were calculated. Eloquent region involved predominantly motor (45%) and language (40%). At first follow up after 3 months permanent neuro-logical deficits (NDs) were noted in 39%. Mild deficits remained in 29% and severe deficits in 10%. Complete tumor removal (CTR) was successfully in 62% of intended cases. Postoperative and 3-month follow up National Institute of Health Stroke Score (NIHSS) showed significantly lower values than preoperatively (p<0.001). 38% cases showed a decreased NIHSS at 3-month, while occurrence was only 14% at 9-12-month follow up. 6/7 patients with mild aphasia recovered after 9-12 months, while motor deficits present at 3-month follow up were persistent in majority of patients. Eastern oncology group functional status (ECOG) significantly decreased by surgery (p < 0.001) in 31% of cases. Between 3-month and 9-12-months follow up no significant improvement was seen. In the multivariable model CTR (p=0.019, OR 31.9), and ECOG>0 (p=0.021, OR 8.5) were independent predictors for permanent postoperative deficit according to NIHSS at 3-month according to multivariable regression model. Patients harboring eloquently located LGG are highly vulnerable for permanent deficits. Almost one third of patients have a permanent reduction of their functional status based on ECOG. Risk of an extended resection has to be balanced with the respective oncological benefit. Especially, patients with impaired pre-operative status are at risk for new permanent deficits. There is a relevant improvement of neurological symptoms in the first year after surgery, especially for patients with slight aphasia.

## Introduction

Lower grade gliomas (LGG) are typically infiltrative and diffuse growing lesions, commonly involving eloquent regions (1–3). Although, slow progressing, they recur unavoidably and undergo malignant transformation (4). Despite better understanding of molecular patterns resulting in the new classification based on isocitrat dehydrogenase (IDH) mutation, surgery remains the main first line treatment (5, 6). A complete fluid-attenuation inversion recovery (FLAIR) or T2 based resection mostly allows for a longer progression free survival and may decrease rate of malignant transformation (7). Gross total resection or even supramaximal resection became an important goal for surgical treatment (1, 8–13). Nevertheless, the aggressive resection might result unintentionally to inferior quality of life (QoL) and compromise daily routines in both private and working spheres (14, 15). This holds true especially for eloquent lesions. Although, there are multicenter retrospective studies suggesting that a volumetric increase of extent of resection leads to an increased survival (5, 16). A deterioration of patients’ functional status apart from reduced QoL might lead to an exclusion from adjuvant treatment resulting in suboptimal outcome (17). Apart from counterbalancing of maximal safe resection and avoidance of neurological and cognitive deterioration, surgeons have to choose from a wide armamentarium of surgical tools various intraoperative imaging devices or mapping techniques at hand (10, 18, 19).

Currently, there are no randomized controlled trials (RCT) or controlled clinical trials (CCT) available on which to base clinical decision making (20). Informed consent is often based on surgeon’s individual experience (20). Incidence of neurological deficits in eloquent location like insular gliomas is often based on retrospective single center data and can thus be underestimated (21). The aim of our study was to evaluate the outcome of patients with eloquently located LGG based on prospective non-selected data from the Log-Glio registry.

## Materials and Methods

### Study Design and Patient Selection

Patients included in the study were prospectively selected from the Log-Glio Registery (clinicaltrials.gov NCT02686229) of patients between November 2016 and May 2021. Primary selection criteria for the registry are patients with a suspected diagnosis of LGG, based upon initial MRI scans. Further inclusion criteria were age over 18 years and signed informed consent. The LoG-Glio registry is a German based multi-center prospective registry with ongoing follow up every 6 month. Currently 13 centers are participating in the registry. Nine centers took part in the current assessment. The detailed study protocol has been described in detail in our earlier publication (22). For the current study only patients with a final histopathological diagnosis of astrocytoma and oligodendroglioma World Health Organization (WHO) grade II and III according to 2016 classification were selected.

Ethical approval was received by the ethic committee of the University of Ulm (Ethikkommission Ulm, No. 201/15). Study was performed in accordance with the Declaration of Helsinki.

### Patient and Tumor Characteristics

Basic demographic data were extracted from the registry. Follow up as evaluated in this study was performed routinely at 3 months after surgery. Motor, speech and visual cortex as well as basal ganglia were considered as eloquent regions based on Brodman anatomical localization. Furthermore, hippocampus, gyrus cinguli and corpus callosum were defined as eloquent regions as well. Mild postoperative neurological deficit was defined as the decrease of 1 grade according to British Medical Research Council or a new or slightly more pronounced aphasia (1 point on National Institute of Health Stroke Score (NIHSS) sub-scale for language). Severe deficit was defined either as worsening of more than 1 grade or severe aphasia (more than 1 point on NIHSS for language). Surgical complications within the first 3 months after surgery apart from neurological deficits were evaluated separately.

As part of the typical treatment regime in Germany in all centers physiotherapy, speech therapy and neuropsychological therapy is offered to all patients during their in hospital stay. Patients suffering from neurological deficits will be offered an in-patient neurological rehabilitation at their discretion. When radiotherapy is recommended, neurological rehabilitation is usually postponed until end of radiotherapy.

### Statistical Analysis

A descriptive assessment was done for demographic data. As part of the explorative assessment we correlated the typical clinical factors calculating Spearman’s ρ (rho) [WHO grade, histology, type of surgical approach, tumor location, awake surgery, sex, recurrent surgery, delay of surgery of more than 3 months after primary diagnosis, use of intraoperative magnetic resonance imaging (iMRI) or intraoperative ultrasound imaging (iUS)]. Correlations were used exploratively thus, it was not corrected for multiple testing.

Chi Square test with Fischer’s exact test was used for binary comparisons. Sign test with Fisher’s exact test was used for related samples. Kruskal-Wallis test was used to test for differences of neurological deficits by center.

We used a binary multivariable regression model to assess influence on presence of a new neurological deficits based on NIHSS at 3-month follow up. Selection of variables included in the model was hypothesis driven and based on previous literature. Variables included in the regression model were type of surgery, recurrent surgery, awake surgery, IDH mutation status, preoperative neurological deficit, preoperative Eastern oncology group functional status (ECOG), use of iMRI, use of iUS, use of intraoperative monitoring (IOM), time to surgery > 3 months, adjuvant treatment and WHO grade. 63 cases entered the multivariable model. 23 were excluded due to missing values in one of the variables. Statistical significance level was set asa two-sided p<0.05. We used SPSS 28.0. (IBM) for calculations.

## Results

### Patients Characteristics

According to the above mentioned criteria 83 patients with complete data sets were selected for the further analysis. Basic demographic data are summarized in **Table 1**. The most common function at risk was motor function (44.6%, n=37) followed by speech (39.8%, n=33). The most common presenting symptoms were seizures (N=47, 56.6%). 54 patients (68.4%) had no restricts in ECOG performance status (0). ECOG status of 1 was found in 5 patients (6.7%). Astrocytomas were more common than oligodendrogliomas. (56.6%, n=47 vs. 42.7% n=35, **Table 1**). 57 of 71 patients with primary surgery (80.3%) had surgery within the first 3 months after primary diagnosis. Median time to surgery was 0 months, and maximum time to surgery was 81 months.

**Table 1:** Patients´ and treatment characteristics.

Variable	N (%)
**Female sex**	37 (44.6)
**Age >60 years**	14 (16.1)
**Oligodendroglioma vs. astrocytoma**	35 (42.7)
**Isocitrat dehydrogenase (IDH) wildtype**	12 (13.8)
**World Health Organisation (WHO) grade III**	24 (27.6)
**O-6-Methylguanin-DNA-Methyltransferase (MGMT) unmethylated**	12 (14.5)
**Tumor location**	
Frontal	43 (51.8)
Parietal	16 (19.3)
Temporal	15 (18.1)
Occipital	1 (1.2)
Other	8 (9.6)
**Hemisphere**	
Left	46 (55.4)
Right	36 (43.4)
Both	1 (1.2)
**Presenting symptoms**	
Seizure	47 (56.6)
Headache	7 (8.4)
Neurological deficit	4 (4.8)
Incidental	9 (10.8)
Others	16 (19.3)
**Preoperative decreased Eastern oncology group score (ECOG) > 0**	25 (28.7)
**Preoperative deficits according to National Institute of Health Stroke Score (NIHSS) >0**	11 (12.6)
**Timing of surgery > 3 months *(italic only primary surgeries)***	22 (27.2), *14 (19.7)*
**Recurrent surgery**	10 (11.5)
**Awake surgery**	19 (21.8)
**Intraoperative monitoring or mapping (IoM)**	66 (75.9)
**Intraoperative ultrasound**	22 (25.3)
**Intraoperative magnetic resonance imaging (MRI)**	35 (40.2)
**Type of surgery**	
Stereotactic biopsy	8 (9.2)
Open biopsy	3 (3.4)
Intended subtotal resection	30 (34.5)
Intended complete tumor resection (CTR)	42 (48.3)
**Complete tumor resection based on radiological criteria (CTR)**	26 (29.9)
**Adjuvant treatment**	
None (wait and scan)	26 (29.9)
Chemotherapy (CT)	4 (4.6)
Radiotherapy (RT)	10 (11.5)
Consecutive CT & RT	17 (19.5)
Combined CT & RT	25 (28.7)

### Characteristics of Surgical Resection

Awake surgery was performed in 19 cases (22.9%) and recurrent surgery was done in 10 cases (11.5%). iMRI was performed in 35 patients (42.2%), while iUS was used in 22 surgeries (26.5%). Intraoperative neuromonitoring (IoM) was applied in 66 cases (79.5%).

### Surgical Complications

Surgical complications apart from new neurological deficits were noted in 14 (16.9%) patients. They are summarized in **Table 2**. Ischemic complications resulted in permanent neurological deficits in four of five cases. All patient had a visible preoperative contact of larger vessels with the tumor. Three patients had insular lesions, one patient a lesion in basal ganglia and one patient suffered from a bifrontal tumor.

**Table 2:** Surgical complications (CSF – cerebrospinal fluid).

Complications	N (83)
Infection	2.4% (2)
CSF Leakage	1.2% (1)
Meningitis	1.2% (1)
Ischemic lesion	6.0% (5)
Hemorrhage	3.6% (3)
Others	2.4& (2)

### New Neurological Deficits

Considering new neurological impairment directly after surgery, 43 (51.8%) patients showed no neurological worsening, while 27 (32.5%) patients had mild new neurological deficit. The remaining 13 (15.7%) patients had a severe postoperative neurological deficit. Four of these patients suffered from ischemic lesions and one patient had a hemorrhage. Patients with mild deficits showed an improvement in 14 (52%) patients,it remained stable in 11 (41%) patients. and decreased in 2 (7%) patients. The differences were significant in Sign test (p=0.004). In patients with severe new deficits, 7 (53.8%) patients improved all others retained severe deficits. These differences were significant as well p=0.016.

At first follow up after 3 months, permanent new postoperative neurological deficits were noted in 32 (38.6%) patients. Mild deficits remained in 24 (28.9%) patients and severe deficits in 8(9.6%). patients.

At first follow up after 3 month NIHSS was decreased in 27 patients compared to preoperative values (37.5%) representing an objective prevalence of new permanent deficits. Concerning NIHSS score, both postoperative and follow up NIHSS showed significantly lower values (p<0.001), while postoperative and follow up NIHSS showed no statistical difference (p=0.213). Comparing preoperative and 1^st^ follow up NIHSS, an improvement was seen only in 5/71 (7%) patients, 27(56%) patients remained stable and 27 (38%) decreased after surgery.

Second follow up between 9 -12 months after surgery was available in 19 of 27 (70%) patients with neurological deterioration after surgery according to NIHSS. We found a significant difference of NIHSS from 3 months follow up to 9-12 months follow up (p<0.001). From 1^st^ to 2^nd^ follow up 13 of 19 (68%) patients improved in NIHSS. Twelve of those showed no deficits according to NIHSS. Patients with permanent deficits at 9-12 months (7) had motor deficits in 6 of 7 (86%) cases. Only one patient with a motor deficit showed an improvement of NIHSS. Six of seven (86%) patients with a mild aphasia (1 point in NIHSS) at 3-month follow up recovered until 9-12-month follow up.

We searched for center effects on change of NIHSS between preoperative score and follow up score using Kruskal-Wallis test and found no significant differences (p=0.966).

### Functional Outcome Based on ECOG Performance Status

**Figure 1:**
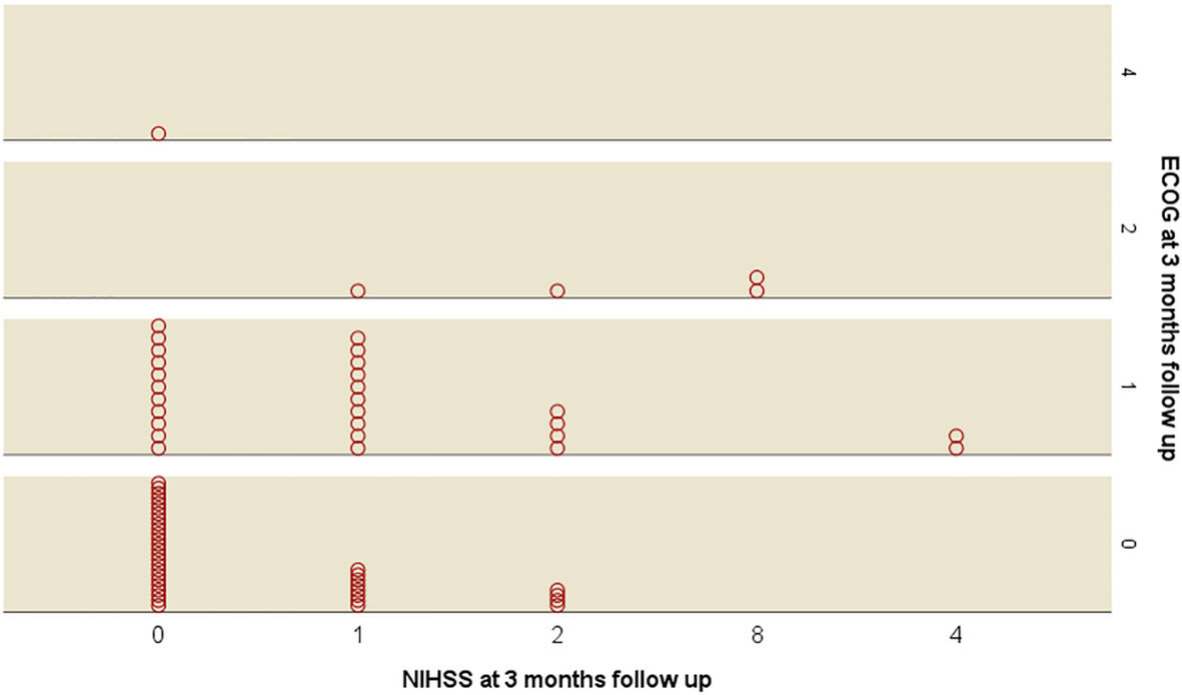
Histogram comparing Eastern Oncology Group (ECOG) score and National institute of health score (NIHSS).

Overall performance status of the patients as documented in ECOG also reflect the above mentioned findings for neurological deficits: At first follow up, ECOG decreased in 22(31%) patients compared to pre-OP. ECOG before surgery was significantly higher if compared to postoperative and follow up ECOG (p<0.001 and p=0.022 respectively). The difference between postoperative and follow up ECOG did not reach statistical difference (p=0.089). Detailed overview is depicted in **Table 3**. Increased NIHSS at follow up correlated significantly with increase ECOG (p=0.003, ρ 0.352). On the other hand, 15 of 41 (36.7%) patients with a normal NIHSS had a decreased ECOG. **Figure 1** shows a histogram comparing ECOG and NIHSS results. Follow up for ECOG between 9-12 months was available in 51 (62%) patients. We assessed all patients, not only patients with decreased ECOG after surgery since a deterioration is also possible following adjuvant treatment. From 1^st^ to 2^nd^ follow up 10 (21%) patients improved, 4 (9%) patients declined and 33 (70%) remained stable. There was no significant difference of 1^st^ to 2^nd^ follow up in ECOG (p=0.180).

**Table 3:** The Eastern Cooperative Oncology Group (ECOG) and National Institute for Stroke Scale (NIHSS) before surgery, at discharge and during follow up.

ECOG	Before surgery (N=79)	At discharge (N=79)	3-month follow up (N=75)	9-12-month follow up (N=51)
0	68.4% (54)	40.5% (32)	54.7% (41)	41.0% (34)
1	26.2% (21)	41.8% (33)	28.7% (29)	16.9% (14)
2	3.6% (3)	15.2% (12)	5.3% (4)	3.6% (3)
3	1.2% (1)	2.5% (2)	0	0
4	0	0	1.2% n (1)	0
5	0	0	0	0
**NIHSS**	**Before surgery (N=75)**	**At discharge (N=69)**	**3-month follow up (N=77)**	**9-12-month follow up (N=51)**
0	85.3% (64)	50.7% (35)	54.5% (42)	86.3% (0)
1	6.7% (5)	26.1% (18)	24.7% (19)	5.9% (3)
2	6.7% (5)	5.8% (4)	11.7% (9)	2.0% (1)
3	0	7.2% (5)	0	2.0% (1)
4	1.3% (1)	1.4% (1)	3.9% (3)	3.9% (2)
>4	0	8.8% (6)	5.2% (4)	0

### Extent of Resection

Complete tumor resection (CTR) was intended in 42 (50.6%). patients. Subtotal resection was planned in 30 (36.1%), cases,extended biopsy in 3 (3.6%) patients and stereotactic biopsy in 8 (9.6%) patients. After surgery assumed CTR by surgeon was noted in 38.6% (n=32) and radiologically confirmed in 31.3% (n=26), so that CTR was successfully in 61.9% of intended cases (N=26/42). When a CTR was intended it was achieved using iMRI in 16 of 22 (73%) patients and, using ultrasound only in one of 8 (12%) patients. iMRI showed a significant correlation with CTR (p=0.010, ρ -0.292) when assessed in all cases. iMRI was used more often when a CTR was intended (22/35, 63%). It was also used for intended STR in 11 of 35 (31%) patients and in open biopsies two of 35 (6%) patients.

**Figure 2:**
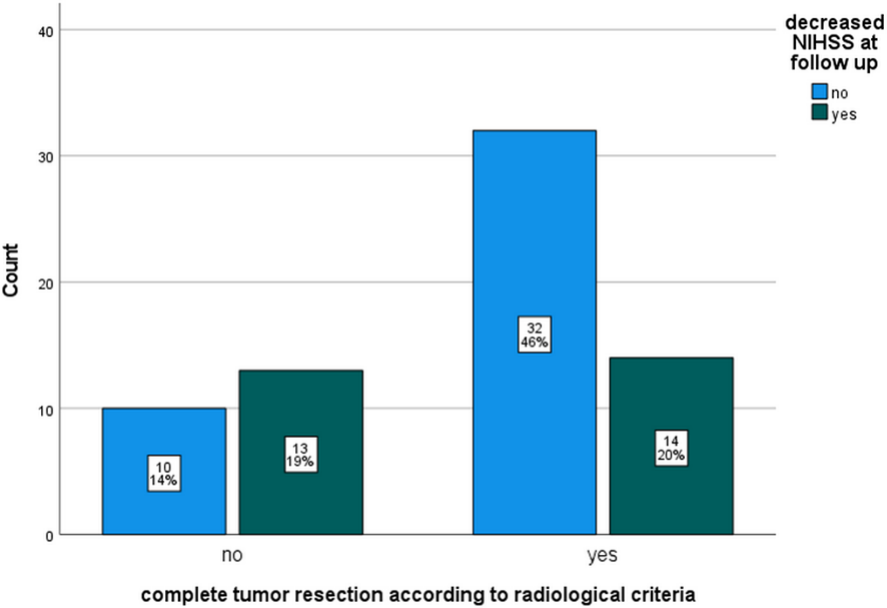
Bar chart comparing proportions of decreased National institute of health score (NIHSS) at 3-month follow up compared to preoperative scores by complete tumor resection (CTR) according to radiological criteria.

After CTR patients had a significantly higher rate of decreased NIHSS at follow up (50% vs.26% p= 0.036 Chi-Square test). **Figure 2** shows a bar chart comparing prevalence of decreased NIHSS at follow up compared to preoperative scores for CTR.

### Influencing Factors on New Permanent Neurological Deficits at 3-Month Follow Up

Presence of a decreased NIHSS score at follow up compared to preoperative data correlated significantly with elevated NIHSS before surgery (p=0.031 ρ –.255), awake surgery (p=0.044, ρ.238), CTR based on radiological imaging (p=0.037, ρ -.252) No significant correlation was found for patient’s age, WHO grade, IDH mutation, type of surgery (stereotactic biopsy (STX) as indicator), tumor location (insular as indicator), recurrent surgery, intraoperative monitoring, intraoperative ultrasound, intraoperative MRI, adjuvant treatment and sex. In patients with primary surgery (n=73) time to surgery > 3 month significantly correlated with decreased NIHSS at follow up (p=0.047, ρ -.255).

We performed a binary multivariable logistic regression for permanent neurological deficits as shown in **Table 4**. CTR (p=0.019, odds ratio (OR) 31.9) and ECOG>0 (p=0.021, OR 11.2) showed a significant influence on new permanent neurological deficits based on NIHSS. Awake surgery showed a tendency towards a significant influence (p=0.060, OR 8.5). IDH mutation, WHO grade, tumor location, IOM, iMRI, iUS, age, type of surgery, adjuvant treatment, time to surgery > 3 months and preoperatively decreased NIHSS showed no significant difference. We also calculated the binary regression model for cases with primary surgery only, because of the interaction of recurrent surgery and time to surgery. We found no relevant differences in the calculation.

**Table 4:** Multivariable binary logistic regression for presence of new permanent neurological deficits at 3 months follow-up after surgery according to National Institute of Health Score.

		B	S.E.		Wald	df	Sig.	Odds Ratio (OR)	95% C.I. for odds ratio	
									Lower	Upper
IDH mutation positive	,381			1,475	,067	1	,796	1,46	,081	26,361
WHO grade III	,422			1,095	,149	1	,700	1,53	,178	13,045
Tumor location (frontal indicator)					1,979	4	,740			
Parietal (1)	-1,678			1,269	1,749	1	,186	,19	,016	2,245
Temporal (2)	-,419			1,134	,136	1	,712	,66	,071	6,075
Insula (3)	-20,514			40192,970	,000	1	1,000	,000	,000	.
Basal ganglia (4)	-,106			1,687	,004	1	,950	,90	,033	24,514
Recurrent surgery	,972			1,536	,400	1	,527	2,64	,130	53,612
Awake surgery	2,141			1,140	3,525	1	,060	8,51	,910	79,534
Intraoperative monitoring	-,372			1,705	,048	1	,827	,69	,024	19,497
Intraoperative ultrasound	1,120			1,092	1,053	1	,305	3,07	,361	26,050
Intraoperative MRI	,185			,956	,037	1	,847	1,20	,185	7,841
Complete tumor resection	3,463			1,471	5,545	1	,019	31,917	1,787	569,731
Age > 60 years (1)	,760			1,239	,377	1	,539	2,149	,189	24,245
Preoperatively impaired ECOG (>0)	2,412			1,044	5,337	1	,021	11,16	1,442	86,398
Preoperative deficits (NHISS >0)	-,379			1,689	,050	1	,822	,68	,025	18,745
Type of surgery (indicator intended gross total resection)					,559	3	,906			
Stereotactic biopsy (1)	-1,107			3,810	,084	1	,771	,33	,000	578,326
Open biopsy (2)	-1,988			3,060	,422	1	,516	,14	,000	55,043
Intended subtotal resection (3)	-1,909			2,947	,419	1	,517	,15	,000	47,800
Adjuvant treatment (indicator not treatment)					3,114	4	,539			
Chemotherapy (CT) (1)	3,811			2,232	2,916	1	,088	45,21	,569	3590,943
Radiotherapy (RT) (2)	,665			1,454	,209	1	,647	1,95	,112	33,642
Consecutive RT & CT (3)	,939			1,200	,612	1	,434	2,56	,244	26,860
Combined RT & CT (4)	,798			1,379	,335	1	,563	2,22	,149	33,166
Time to surgery > 3 months from primary diagnosis	-,658			1,094	,362	1	,548	,528	,061	4,423
Constant	-1,966			2,392	,675	1	,411	,14		

IDH, Isocitrat dehydrogenase; WHO, World Health Organization; MRI, Magnetic resonance imaging; NHISS, National Institute of Health Stroke Score; ECOG, Eastern Oncology Group Status Score.

### Subgroup Assessment

#### Awake Surgery

Based on our finding we further assessed the subgroup of the 19 (22%) patients with awake surgery: rate of preoperative deficits was lower compared to the other patients’ (5% vs. 16%), while rate of insular involvement was slightly higher (11% vs. 8%). No impairment of preoperative ECOG was found less often in awake operated patients (58% vs. 67%). Rate of intended CTR was similar to asleep operated patients (47% vs. 52%).

#### Surgery > 3 Months After Primary Diagnosis

In patients who underwent surgery later than 3 months after the primary diagnosis, WHO grade II was more common (n=13, 93% vs. 38, 67%). They had slightly more often preoperative deficits in NIHSS (3, 21% vs. 7, 14%) and less often an impaired ECOG (>0) (3, 21% vs. 20, 37%). Majority (7, 54% vs. 7, 12%) of the delayed surgeries were accidental findings. Rate of epileptic seizures as presenting symptoms was lower in patients who underwent surgery after 3 months (5, 39% vs. 36, 63%). Distribution of tumor location as well as functional involvement were relatively similar (e.g. language 43% vs. 42%).

#### Stereotactic Biopsy

One of eight (13%) patient after STX had a decrease of NIHSS at follow up. All others had no new neurological deficits. This is the lowest rate compared to all other surgical approaches (open biopsy 1/3, 33%; intended subtotal resection 10/29, 35%; intended complete tumor resection 15/34, 44%). Patients after STX were slightly older than after other types of surgery: 3/5 (38%) vs. 12/75 (16%) were >60 years old. The preoperative ECOG >0 was lower in these patients, too (3/8 (38%) vs. 22/75 (29%). The occurrence of preoperatively impaired NIHSS was similar to the other types of surgery (1/8, 12.5% vs.10/75, 13.3%).

## Discussion

Lower grade gliomas remain challenging neoplasms, since they affect typically younger patients and commonly infiltrate eloquent regions (2, 4). Tumor integration in neuronal networks may often limit extend of resection (23). There might even be a potential role of glioma’s molecular subtype influencing pathway disruption or displacement (24).

We have performed a detailed evaluation of eloquently located diffuse LGGs based on Log-Glio registry and focused on clinical outcome. Interestingly, despite functional intraoperative monitoring, we found relatively high number of patients with persistent new neurological deficit 3 months after surgery. Overall, almost half of the patients show new neurological deficits right after surgery. Both severe and mild neurological deficits show an improvement in half of these patients. Yet, around one third of patients permanently deteriorate both in neurological functions and in their daily life according to ECOG performance status. Predictive for permanent deficits was an impaired preoperative ECOG and a complete tumor resection in multivariable regression. Our data show that despite improved neuromonitoring and surgical techniques, risk for a permanent neurological deficit is high and improvement within the first 3 months is limited. This holds true, especially if a progressive resection aiming for a CTR is performed. The question arises; are mild neurological impairments justified if CTR is achieved? CTR has been shown to be an independent predictor for longer overall survival and even small tumor remnants could result in inferior survival (5, 16). From our perspective this question may only be answered for each individual patient. Our data may serve as a basis for patients’ informed consent before surgery to discuss potential risks and include them in decision making for surgical strategy. According to actual literature, the reported permanent deficits after glioma surgery range between 2-24% including both motor and speech deficits (5, 9, 12). Our series provides prospective multicenter data including only eloquent lesions. Most cited studies were monocentric and did not use clinical scores like NIHSS for detect deficits and hence may underestimate occurrence of postoperative deficits. This suggests that, the wide range of 2-24% of deficits might be more likely in the upper level when an unselected series is assessed. We found a prevalence of 38% at 3-month follow up. However, more than two-third of the patients improved within the first year of surgery. Especially, patients with a slight aphasia showed a good prognosis. In our series of eloquent tumors rate of intended CTR was relatively high with 48%, also intraoperative imaging was used in the majority of cases, suggesting a rather progressive approach compared to a slightly older series from the French glioma network with a CTR of only 12% including also non-eloquent lesions (25).

Surprisingly, even though only eloquent lesions were selected in our series in 20% of cases no use of IoM was reported which may bias our results. The prevalence of IoM in our series is higher than in the above cited contemporary study by Munkvold et al. (26) (76% vs. 58%). Given that only eloquent lesions were approached, from our point of view all patients should be operated using IoM. Even though, this finding is not supported by the results of the multivariable model. We hypothesize that, similar to awake surgery, with increasing risk of surgery, more likely IoM is used. Hence, protective effects may be leveled statistically.

Timing of surgery seems to play a role also in our data. There was a correlation of permanent new deficits in NIHSS and time to surgery >3month for primary surgeries. In the descriptive data no greater differences were found between patients operated early and patients watched and scanned except, that mostly accidental findings and tumors most likely being a WHO grade II lesion were watched for a longer time. This correlates with the current German management guidelines for glioma (27). Our data shows a slightly lower but relatively similar number of watch and scan than a current large Scandinavian series with 17% of patients (26). The authors have not provided the outcome data of these patients, so far. Based on the earlier findings from Jakola et al., being still the best evidence favoring an early surgical resection, one would expect lower occurrences of watch and scan (28). In our multivariate model ‘delayed’ surgery independently does not show a significant influence on new permanent deficits, but impaired preoperative ECOG did. From our point of view, it is important to avoid new deficits before surgery since it may mean that plasticity and redundancy of networks is already consumed by the tumor growth. Higher preoperative ECOG is a negative predictor factor for a good functional outcome, our data underlines the importance of an early resection before tumor progress results in a functional impairment.

Awake surgery is a gold standard for resection of eloquent located tumors adjacent to or in speech-eloquent cortical areas or fiber tracts (29, 30). However, awake surgery could be interpreted as a potential risk factor for permanent deficit according to our data. This result is limited by the relatively low number of awake surgeries in our series. Further, one might assume a selection bias for these patients since larger and more eloquently located tumors might more likely be operated awake. Compared to patients who had surgery asleep the proportion of preoperative deficits is relatively similar to patients operated awake, as is the rate of tumors involving the insula. On the other hand, preoperative ECOG is impaired more often in these patients reflecting the immense burden of impaired language function for patients. We interpret the potential association of awake surgery with permanent deficits not as a risk factor and we warn to draw the conclusion that asleep surgery is more protective. In direct comparison, current literature shows a superiority in extent of resection and occurrences of neurological deficits of awake to asleep surgery as reported by a current meta-analysis (21). Our data rather demonstrate the risk of operating language eloquent tumors, even when using the awake technique, which should be discussed with patients undergoing surgery in this area. Especially, patients with preoperative deficits seem to be at higher risk for permanent impairments as also found in other series (31). Our series, provides data 3 months after surgery which represents the typical reported time point for neurological deficits also after awake surgery (21, 32). Short term language deficits usually recover during this period (33). The proportion of long-term improvement of language deficits still present at 3 months was reported relatively low. In our series we found that especially slight aphasic deficits have a high probability of improvement within the first year (13, 34). Motor deficits had a more unfavorable outcome. The data of 9-12 months follow up was only available for 2/3^rd^ of patients with new deficits at first follow-up and for a better comparability to previously published data we calculated the regression models with the first follow up only. The remarkable occurrence of recovery in patients with aphasia may be biased by the detection using NIHSS. A false negative rate of 9% was reported by Grönberg et al. for stroke patients (35). Especially, a subtle anomic aphasia can remain undetected in NIHSS and may relevantly impair patients’ daily routines or return to work. Our results regarding ECOG also support this theory: Functional status based on ECOG showed a relatively stable course after surgery. Patients did not recover in overall function as they do on neurological deficits. Further patient reported outcome data like health related quality of life are needed to further address this issue.

In our study we did not find an influence of tumor biology as suggested by Young et al. (24). The authors showed an association of intra-tumoral function and pathway infiltration to molecular subtype of tumor. Whether it also influences surgical outcome has not been shown so far. In our series, both WHO grade and IDH mutations were also not influencing occurrence of permanent deficits. Most likely, effects are subtle and larger series will be needed to further address this question.

Intraoperative imaging like intraoperative MRI and intraoperative ultrasound were shown to increase extent of resection, and are widely used by European neurosurgeons as also in the participating centers in our study (10). Yet, in this eloquent series proportions of CTR are lower than previously published for intraoperative imaging (36–38) since resection is obviously limited by function even in cases in which a CTR was deemed feasible preoperatively. Intraoperative imaging like iMRI was applied more often in patients with an intended CTR. Yet, while CTR highly correlated with permanent deficits and also was an independent predictor for them, intraoperative imaging did not correlate with new permanent neurological deficits in our series. One of the reasons could be that in all cases iMRI was used, it was combined with IoM, for iUS is was slightly lower with 82%. Further, an intraoperative visualization of individual anatomic structures after relevant brain shift may also increase safety. However, no evidence for this hypothesis can be found in the current data.

Interestingly, recurrent surgery showed no significantly higher risk of deficits. One explanation may be the relatively low number of cases in our series. Regarding recurrent surgery, the concept of multiply staged approaches as proposed by Duffau et al. (34) may be a relevant strategy. Cortical reorganization in relation to function by neuroplasticity may increase safety in a second surgical approach. A detailed preoperative functional imaging by functional or resting state MRI, integration of connectomics and/or non-invasive cortical mapping may foster thorough preoperative evaluation in these patients and may increase safety of resection during surgery (39–42). Further studies are needed to evaluate influence of this preoperative data on surgical outcome. Another future perspective is to study patient reported outcome measures as health-related quality of life or supportive care need to better understand how fine cognitive difficulties or motor deficits influence patients’ daily life or occupational situation.

### Limitations

Our assessment is based on prospective unselected data from a multicenter registry. Hence, different surgical strategies and therapeutic strategies are entered in the assessment. This unselected overview is a strength of this assessment as it reflects routine procedures and it is more likely comparable other neurosurgical centers than single center data but, it also limits the statistical assessments and the power of our subsequent analyses.

Data of our study likely reflects the current German treatment situation in neuro-oncological centers. It may not be transferable worldwide for low grade glioma surgery.

Even if our data originate from prospectively collected dataset including NIHSS and ECOG, it does not replace detailed neuropsychological tests. Especially cognitive and language function are underrepresented in NIHSS and can only indirectly be measured in ECOG score. Functioning scores like ECOG represent an external view and may not adequately reflect everyday life of affected patients. The definition of eloquent located tumors is based on respective surgeon’s assessment and does not necessarily correlate with intraoperative functional borders. Hence lesions with near eloquent location and eloquent location according to Sawaya’s classification are mixed in this series (43). Exact tumor location can be defined more detailed using Broca’s areas and central nuclei. In the LoG-Glio prospective registry a lobular classification is used. CTR was defined by local radiologist of certified oncological centers. Yet, no central reading was performed. Further, no volumetric assessment of residual tumor was performed. The number of parameters considered in the analysis, with respect to the population size is too large to warrant a sufficient statistical power for the negative findings of this study. Hence, potential influence of surgical techniques or other clinical markers may be missed.

Postoperative and follow up rehabilitation including speech therapy and physiotherapy may relevantly improve neurological outcome. In the LoG-Glio registry no data is included whether patients attended these programs or not. However, since all patients in the participation centers have free-access to all option for neurological rehabilitation as mentioned in material and methods, most likely it was utilized by patients with deficits.

## Conclusions

Patients harboring eloquently located LGG are highly vulnerable for permanent deficits. Almost one third of patients have a permanent reduction of their functional status based on ECOG. Risk of an extended resection has to be balanced with the respective oncological benefit. Especially, patients with impaired pre-operative status are at risk for new permanent deficits. There is a relevant improvement of neurological symptoms in the first year after surgery, especially for patients with slight aphasia.

## Data Availability Statement

The original contributions presented in the study are included in the article/supplementary material, further inquiries can be directed to the corresponding author/s.

## Ethics Statement

Ethical approval was received by the ethic committee of the University of Ulm (Ethikkommission Ulm, No. 201/15). The patients/participants provided their written informed consent to participate in this study.

## Author Contributions

Conceptualization, JC, CW, AP, GN, DR. Methodology, JC, AP. Formal analysis, JC. Investigation, JC, JO, MU, CR, KK, MN, M-TF, RG, ML. Resources, CW, VR, MC, OG, PV, MT, DR. Data curation, JC. Writing—original draft preparation, AP, JC. Writing—review and editing, all contributing authors. All authors have read and agreed to the published version of the manuscript.

## Conflict of Interest

The authors declare that the research was conducted in the absence of any commercial or financial relationships that could be construed as a potential conflict of interest.

## Publisher’s Note

All claims expressed in this article are solely those of the authors and do not necessarily represent those of their affiliated organizations, or those of the publisher, the editors and the reviewers. Any product that may be evaluated in this article, or claim that may be made by its manufacturer, is not guaranteed or endorsed by the publisher.

## References

[1] Ius T Isola M Budai R Pauletto G Tomasino B Fadiga L. Low-Grade Glioma Surgery in Eloquent Areas: Volumetric Analysis of Extent of Resection and Its Impact on Overall Survival. A Single-Institution Experience in 190 Patients. J Neurosurg (2012) 117:1–14. doi: 10.3171/2012.8.jns12393 23039150

[2] Duffau H Capelle L. Preferential Brain Locations of Low-Grade Gliomas. Cancer (2004) 100:2622–6. doi: 10.1002/cncr.20297 15197805

[3] Jakola AS Unsgård G Myrmel KS Kloster R Torp SH Lindal S. Low Grade Gliomas in Eloquent Locations - Implications for Surgical Strategy, Survival and Long Term Quality of Life. PloS One (2012) 7(12):e51450. doi: 10.1371/journal.pone.0051450 23251537 PMC3519540

[4] Pouratian N Schiff D. Management of Low-Grade Glioma. Curr Neurol Neurosci Rep (2010) 10:224–31. doi: 10.1007/s11910-010-0105-7 PMC2857752 20425038

[5] Wijnenga MMJ French PJ Dubbink HJ Dinjens WNM Atmodimedjo PN Kros JM. The Impact of Surgery in Molecularly Defined Low-Grade Glioma: An Integrated Clinical, Radiological, and Molecular Analysis. Neuro-Oncology (2018) 20:103–12. doi: 10.1093/neuonc/nox176 PMC5761503 29016833

[6] Louis DN Perry A Wesseling P Brat DJ Cree IA Figarella-Branger D. The 2021 WHO Classification of Tumors of the Central Nervous System: A Summary. Neuro-Oncology (2021) 23:1231–51. doi: 10.1093/neuonc/noab106 PMC8328013 34185076

[7] Snyder LA Wolf AB Oppenlander ME Bina R Wilson JR Ashby L. The Impact of Extent of Resection on Malignant Transformation of Pure Oligodendrogliomas. J Neurosurg (2014) 120:309–14. doi: 10.3171/2013.10.JNS13368 24313617

[8] Pallud J Varlet P Devaux B Geha S Badoual M Deroulers C. Diffuse Low-Grade Oligodendrogliomas Extend Beyond MRI-Defined Abnormalities. Neurology (2010) 74:1724–31. doi: 10.1212/WNL.0b013e3181e04264 20498440

[9] Sanai N Berger MS. Glioma Extent of Resection and Its Impact on Patient Outcome. Neurosurgery (2008) 62:753–64-discussion 264-. doi: 10.1227/01.neu.0000318159.21731.cf 18496181

[10] Coburger J Merkel A Scherer M Schwartz F Gessler F Roder C. Low-Grade Glioma Surgery in Intraoperative Magnetic Resonance Imaging: Results of a Multicenter Retrospective Assessment of the German Study Group for Intraoperative Magnetic Resonance Imaging. Neurosurgery (2016) 78:775–86. doi: 10.1227/neu.0000000000001081 26516822

[11] Keles GE Lamborn KR Berger MS. Low-Grade Hemispheric Gliomas in Adults: A Critical Review of Extent of Resection as a Factor Influencing Outcome. J Neurosurg (2001) 95:735–45. doi: 10.3171/jns.2001.95.5.0735 11702861

[12] McGirt MJ Chaichana KL Attenello FJ Weingart JD Than K Burger PC. Extent of Surgical Resection Is Independently Associated With Survival in Patients With Hemispheric Infiltrating Low-Grade Gliomas. Neurosurgery (2008) 63:700-7-author reply 707-8. doi: 10.1227/01.neu.0000325729.41085.73 18981880

[13] Duffau H Taillandier L. New Concepts in the Management of Diffuse Low-Grade Glioma: Proposal of a Multistage and Individualized Therapeutic Approach. Neuro-Oncology (2015) 17(3):332–42. doi: 10.1093/neuonc/nou153 PMC4483091 25087230

[14] Reijneveld JC Taphoorn MJB Coens C Bromberg JEC Mason WP Hoang-Xuan K. Health-Related Quality of Life in Patients With High-Risk Low-Grade Glioma (EORTC 22033-26033): A Randomised, Open-Label, Phase 3 Intergroup Study. Lancet Oncol (2016) 17:1533–42. doi: 10.1016/s1470-2045(16)30305-9 27686943

[15] Duffau H Mandonnet E. The “Onco-Functional Balance” in Surgery for Diffuse Low-Grade Glioma: Integrating the Extent of Resection With Quality of Life. Acta Neurochirurgica (2013) 155:951–7. doi: 10.1007/s00701-013-1653-9 23447053

[16] Scherer M Ahmeti H Roder C Gessler F Jungk C Pala A. Surgery for Diffuse WHO Grade II Gliomas: Volumetric Analysis of a Multicenter Retrospective Cohort From the German Study Group for Intraoperative Magnetic Resonance Imaging. Neurosurgery (2020) 86(1):E64–74. doi: 10.1093/neuros/nyz397 31574147

[17] Bent MJvd Afra D Witte O Hassel MB Schraub S Hoang-Xuan K. Long-Term Efficacy of Early Versus Delayed Radiotherapy for Low-Grade Astrocytoma and Oligodendroglioma in Adults: The EORTC 22845 Randomised Trial. Lancet (London England) (2005) 366:985–90. doi: 10.1016/s0140-6736(05)67070-5 16168780

[18] Jaber M Wölfer J Ewelt C Holling M Hasselblatt M Niederstadt T. The Value of 5-Aminolevulinic Acid in Low-Grade Gliomas and High-Grade Gliomas Lacking Glioblastoma Imaging Features: An Analysis Based on Fluorescence, Magnetic Resonance Imaging, 18F-Fluoroethyl Tyrosine Positron Emission Tomography, and Tumor Molecular Factors. Neurosurg (2016) 78:401–11-discussion 411. doi: 10.1227/neu.0000000000001020 PMC4747980 26366972

[19] Stummer W Novotny A Stepp H Goetz C Bise K Reulen HJ. Fluorescence-Guided Resection of Glioblastoma Multiforme by Using 5-Aminolevulinic Acid-Induced Porphyrins: A Prospective Study in 52 Consecutive Patients. J Neurosurg (2000) 93:1003–13. doi: 10.3171/jns.2000.93.6.1003 11117842

[20] Jiang B Chaichana K Veeravagu A Chang SD Black KL Patil CG. Biopsy Versus Resection for the Management of Low-Grade Gliomas. Cochrane Database Syst Rev (2017) 4:CD009319. doi: 10.1002/14651858.cd009319.pub3 28447767 PMC6478300

[21] Bu L-H Zhang J Lu J-F Wu J-S. Glioma Surgery With Awake Language Mapping Versus Generalized Anesthesia: A Systematic Review. Neurosurg Rev (2021) 44:1997–2011. doi: 10.1007/s10143-020-01418-9 33089447

[22] Pala A Nadji-Ohl M Faust K Rückriegel S Roder C Brelie Cvd. Epidemiological and Biological Disease Profile as Well as Clinical Outcome in Patients With Low-Grade Gliomas: The Log-Glio Project. J Neurol Surg A Cent Eur Neurosurg (2019). doi: 10.1055/s-0039-1693650 31550737

[23] Picart T Herbet G Moritz-Gasser S Duffau H. Iterative Surgical Resections of Diffuse Glioma With Awake Mapping: How to Deal With Cortical Plasticity and Connectomal Constraints? Neurosurgery (2019) 85:105–16. doi: 10.1093/neuros/nyy218 30010996

[24] Young JS Gogos AJ Morshed RA Hervey-Jumper SL Berger MS. Molecular Characteristics of Diffuse Lower Grade Gliomas: What Neurosurgeons Need to Know. Acta Neurochir (2020) 162:1929–39. doi: 10.1007/s00701-020-04426-2 32472378

[25] Capelle L Fontaine D Mandonnet E Taillandier L Golmard JL Bauchet L. Spontaneous and Therapeutic Prognostic Factors in Adult Hemispheric World Health Organization Grade II Gliomas: A Series of 1097 Cases: Clinical Article. J Neurosurg (2013) 118:1157–68. doi: 10.3171/2013.1.jns121 23495881

[26] Munkvold BKR Solheim O Bartek J Corell A de DE Gulati S. Variations in the Management of Diffuse Low-Grade Gliomas—A Scandinavian Multicenter Study. Neuro-Oncol Pract (2021) 8:706–17. doi: 10.1093/nop/npab054 PMC8579093 34777840

[27] Bendszus M Goldbrunner R Grosu A Hattingen E Hau P Herrlinger U. Gliome, S2k-Leitlinie, 2021. In: Deutsche Gesellschaft FöR Neurologie (Hrsg.), Leitlinien FöR Diagnostik Und Therapie in Der Neurologie. Heidelberg Germany: Deutsche Gesellschaft für Neurologie (2021).

[28] Jakola AS. Comparison of a Strategy Favoring Early Surgical Resection vs a Strategy Favoring Watchful Waiting in Low-Grade. JAMA: J Am Med Assoc (2012) 308(18):1881–8. doi: 10.1001/jama.2012.12807 23099483

[29] Moritz-Gasser S Duffau H Yordanova YN. Awake Surgery for WHO Grade II Gliomas Within “Noneloquent” Areas in the Left Dominant Hemisphere: Toward a “Supratotal” Resection. Clin Article (2011) 115:232–9. doi: 10.3171/2011.3.JNS101333 21548750

[30] Maldaun MVC Khawja SN Levine NB Rao G Lang FF Weinberg JS. Awake Craniotomy for Gliomas in a High-Field Intraoperative Magnetic Resonance Imaging Suite: Analysis of 42 Cases. J Neurosurg (2014) 121:1–8. doi: 10.3171/2014.6.jns132285 25105702

[31] Serletis D Bernstein M. Prospective Study of Awake Craniotomy Used Routinely and Nonselectively for Supratentorial Tumors. J Neurosurg (2007) 107:1–6. doi: 10.3171/jns-07/07/0001 17639865

[32] Eseonu CI Rincon-Torroella J ReFaey K Lee YM Nangiana J Vivas-Buitrago T. Awake Craniotomy vs Craniotomy Under General Anesthesia for Perirolandic Gliomas: Evaluating Perioperative Complications and Extent of Resection. J Neurosurg (2017) 81:481–9. doi: 10.1093/neuros/nyx023 28327900

[33] Caverzasi E Hervey-Jumper SL Jordan KM Lobach IV Li J Panara V. Identifying Preoperative Language Tracts and Predicting Postoperative Functional Recovery Using HARDI Q-Ball Fiber Tractography in Patients With Gliomas. J Neurosurg (2015) 125:33–45. doi: 10.3171/2015.6.jns142203 26654181

[34] Duffau H Robles SG Gatignol P Lehéricy S. Long-Term Brain Plasticity Allowing a Multistage Surgical Approach to World Health Organization Grade II Gliomas in Eloquent Areas. J Neurosurg (2008) 109:615–24. doi: 10.3171/JNS/2008/109/10/0615 18826347

[35] Grönberg A Henriksson I Lindgren A. Accuracy of NIH Stroke Scale for Diagnosing Aphasia. Acta Neurol Scand (2020) 143:375–82. doi: 10.1111/ane.13388 PMC7985870 33368189

[36] Zhang G Li Z Si D Shen L. Diagnostic Ability of Intraoperative Ultrasound for Identifying Tumor Residual in Glioma Surgery Operation. Oncotarget (2017) 8:73105–14. doi: 10.18632/oncotarget.20394 PMC5641196 29069853

[37] Senft C Bink A Franz K Vatter H Gasser T Seifert V. Intraoperative MRI Guidance and Extent of Resection in Glioma Surgery: A Randomised, Controlled Trial. Lancet Oncol (2011) 12:997–1003. doi: 10.1016/s1470-2045(11)70196-6 21868284

[38] Pala A König R Hlavac M Wirtz CR Coburger J. Does the Routine Use of Intraoperative MRI Prolong Progression Free Survival in Low-Grade Glioma Surgery? A Retrospective Study. Innovative Neurosurg (2015) 3:109–8. doi: 10.1515/ins-2015-0003

[39] Durner G Pala A Federle L Grolik B Wirtz CR Coburger J. Comparison of Hemispheric Dominance and Correlation of Evoked Speech Responses Between Functional Magnetic Resonance Imaging and Navigated Transcranial Magnetic Stimulation in Language Mapping. J Neurosurg Sci (2019) 63:106–13. doi: 10.23736/s0390-5616.18.04591-5 30259722

[40] Burks JD Bonney PA Conner AK Glenn CA Briggs RG Battiste JD. A Method for Safely Resecting Anterior Butterfly Gliomas: The Surgical Anatomy of the Default Mode Network and the Relevance of Its Preservation. J Neurosurg (2016) 126:1795–811. doi: 10.3171/2016.5.jns153006 PMC9473322 27636183

[41] Sollmann N Kelm A Ille S Schröder A Zimmer C Ringel F. Setup Presentation and Clinical Outcome Analysis of Treating Highly Language-Eloquent Gliomas *via* Preoperative Navigated Transcranial Magnetic Stimulation and Tractography. Neurosurg Focus (2018) 44:E2. doi: 10.3171/2018.3.focus1838 29852769

[42] Coburger J Musahl C Henkes H Horvath-Rizea D Bittl M Weissbach C. Comparison of Navigated Transcranial Magnetic Stimulation and Functional Magnetic Resonance Imaging for Preoperative Mapping in Rolandic Tumor Surgery. Neurosurg Rev (2012) 36:65–75; discussion 75-6. doi: 10.1007/s10143-012-0413-2 22886323

[43] Sawaya R Hammoud M Schoppa D Hess KR Wu SZ Shi W-M. Neurosurgical Outcomes in a Modern Series of 400 Craniotomies for Treatment of Parenchymal Tumors. Neurosurgery (1998) 42:1044–55. doi: 10.1097/00006123-199805000-00054 9588549

